# Promoting hand hygiene in a chemotherapy day center: the role of a robot

**DOI:** 10.1186/s13756-024-01510-5

**Published:** 2024-12-21

**Authors:** Shuk-Ching Wong, Stephen Chun-Yat Ip, Monica Oi-Tung Kwok, Crystal Yuen-Ki Siu, Jonathan Hon-Kwan Chen, Simon Yung-Chun So, Kelvin Hei-Yeung Chiu, Kwok-Keung Yuen, Vincent Chi-Chung Cheng

**Affiliations:** 1 Infection Control Team, Queen Mary Hospital, Hong Kong West Cluster, Pokfulam, Hong Kong Special Administrative Region, China; 2 Department of Microbiology, Queen Mary Hospital, Pokfulam, Hong Kong Special Administrative Region, China; 3 School of Nursing, Li Ka Shing Faculty of Medicine, The University of Hong Kong, Pokfulam, Hong Kong Special Administrative Region, China; 4 Department of Clinical Oncology, Queen Mary Hospital, Pokfulam, Hong Kong Special Administrative Region, China

**Keywords:** Hand hygiene, Promotion, Chemotherapy day center, Robot

## Abstract

**Background:**

Hand hygiene is a critical component of infection prevention in healthcare settings. Innovative strategies are required to enhance hand hygiene practices among patients and healthcare workers (HCWs).

**Methods:**

This study was conducted at the Chemotherapy Day Center of Queen Mary Hospital, Hong Kong. It comprised three phases: phase 1 involved observational audits of hand hygiene practices among patients and HCWs by infection control nurse (ICN); phase 2 included the installation of 53 pressure sensors on alcohol-based hand rub (AHR) bottles at designated sites to monitor usage; phase 3 introduced the robot named Temi Medic to promote hand hygiene through video broadcasts at strategic locations in the center. The mean counts of pressure sensor-equipped AHR per 100 attendances per day (hereafter referred to as the mean count) across phases 2 and 3 were analyzed.

**Results:**

A total of 2580 patient attended the center from April to September 2023. The ICN observed a significant increase in hand hygiene practices among patients at the entrance and reception area, rising from phase 1 (0.2%, 1/583) and phase 2 (0.5%, 3/656) to phase 3 (5.0%, 33/654) (*p* < 0.001). Meanwhile, the overall hand hygiene compliance among HCWs was 74.1% (1341/1810) throughout the study period. From phase 2 to phase 3, the mean counts of 7 AHR bottles designated for patient use (P1–P7) significantly increased (35 ± 17 vs. 64 ± 24, *p* < 0.001), as did the 33 AHR bottles shared by both patients and HCWs (207 ± 104 vs. 267 ± 113, *p* = 0.027). In contrast, there was no significant change in the mean count among the 13 AHR bottles designated for HCWs (H1–H13). The mean count of H1–H13 was significantly higher than that of P1–P7 throughout phases 2 and 3 (214 ± 93 vs. 49 ± 25, *p* < 0.001), indicating a 4.4-fold difference.

**Conclusions:**

While HCWs maintained stable hand hygiene compliance, the introduction of the robot significantly improved hand hygiene practices among patients in the chemotherapy day center. This underscores the importance of integrating technology into routine practices to promote infection prevention and control in healthcare settings.

**Supplementary Information:**

The online version contains supplementary material available at 10.1186/s13756-024-01510-5.

## Introduction

Hand hygiene is a critical component of infection prevention in healthcare settings. The World Health Organization (WHO) emphasizes that proper hand hygiene can significantly reduce the transmission of healthcare-associated infections, which pose serious risks to patients, especially those undergoing cancer treatment [1]. Patients undergoing chemotherapy frequently present with compromised immune systems, rendering them especially susceptible to infections. Consequently, it is crucial to ensure that these patients are informed about and adhere to proper hand hygiene practices to safeguard their health and facilitate recovery. However, despite established guidelines and the recognized importance of hand hygiene, compliance rates among healthcare workers (HCWs) and patients often fall short of recommended levels. This gap highlights the need for innovative strategies to enhance hand hygiene practices in healthcare settings.

Recent technological advancements have introduced new opportunities for promoting health behaviors, particularly through the integration of robotics in healthcare environments. A systematic review indicates that robotic technologies can significantly improve adherence to hand hygiene protocols among HCWs. However, widespread adoption has faced challenges, including cost and user acceptance [2]. One noteworthy example is the humanoid robot DAVE, which has demonstrated success in improving hand hygiene adherence in hospital settings by engaging both patients and staff through social interaction and reminders [3].

Here, we explore the use of a robotic system to promote hand hygiene practices in a chemotherapy day center. Our objective is to evaluate the effectiveness of this robotic intervention in hand hygiene practice. By leveraging technology in routine practices, we aim to create a safer healthcare environment that prioritizes infection prevention and control.

## Material and methods

### Setting

**Fig. 1:**
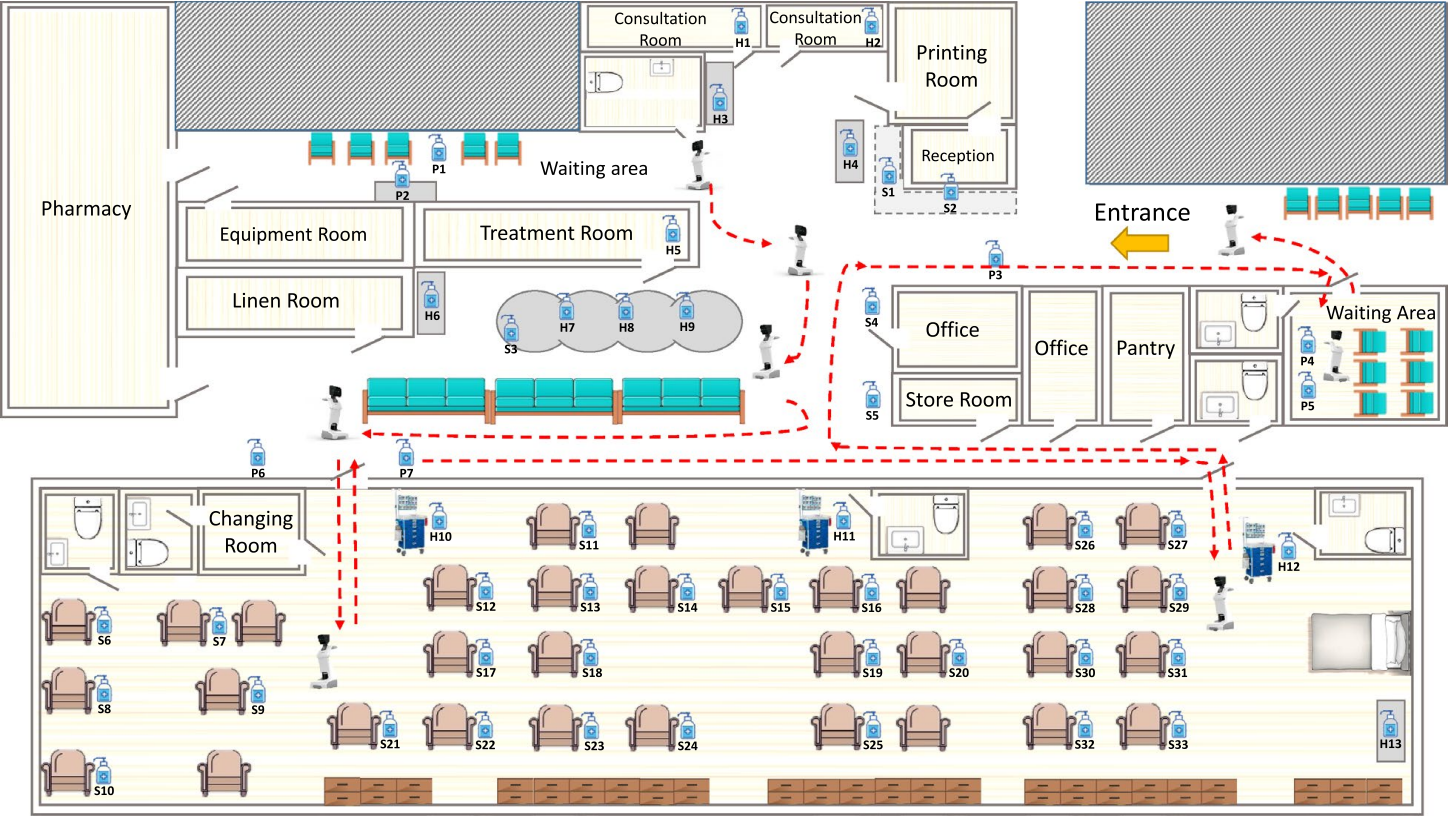
Distribution of pressure sensor-equipped alcohol-based hand rubs and robot path in the chemotherapy day center. *Note* P1–P7 represent seven pressure sensor-equipped alcohol-based hand rub bottles designated for patient use. P1, P2, P4, and P5 are located in the waiting areas of the chemotherapy day center. P3 is situated at the center’s entrance, while P6 and P7 are positioned at the entrance of the chemotherapy administration area. S1 to S33 represent 33 pressure sensor-equipped alcohol-based hand rub bottles shared by patients and healthcare workers. S1 and S2 are located in the reception area. S3 is placed in the nursing station designated for intravenous catheter insertion for receiving chemotherapeutic agents. S4 and S5 are located in the corridor of the waiting area. S6 to S 33 are located inside the chemotherapy administration area. H1–H13 represent 13 pressure sensor-equipped alcohol-based hand rub bottles designated for healthcare workers. H1 and H2 are in the consultation room, H3 and H4 are on the table for blood pressure monitoring, H5 is in the treatment room, and H6 is adjacent to the nursing station. H7–H9 are located in the nursing station for intravenous catheter insertion, H10–H12 are on the medication administration cart in the chemotherapy administration area, and H13 is in the nursing station within the chemotherapy administration area. A robot named Temi Medic (Temi) was programmed to operate in the corridor from 9:00 AM to 10:00 AM, coinciding with patient registration at the entrance reception area. It moved around during three different time intervals: 11:00 AM to 12:00 PM, 1:00 PM to 2:00 PM, and 3:00 PM to 4:00 PM. During these intervals, Temi stopped at seven designated spots (excluding the reception area at the entrance), including the waiting areas, reception area, and chemotherapy administration area, along a specified path, as indicated by the dotted line, to promote hand hygiene by broadcasting videos at these locations

This study was conducted at the Chemotherapy Day Center, Department of Clinical Oncology, Queen Mary Hospital, a tertiary referral center with a capacity of approximately 1700 beds within the Hong Kong West Cluster, under the governance of the Hospital Authority [4]. The Department of Oncology offers comprehensive anti-cancer therapies including chemotherapy, radiotherapy, and palliative care. Chemotherapy services are provided in the oncology ward for inpatients, as well as in the chemotherapy day center for outpatients. The center operates with 15 HCWs from Monday to Saturday, closing on Sundays, public holidays, or during adverse weather conditions such as tropical cyclones. It occupies a converted standard ward of approximately 570 square feet, which includes a reception area, waiting area, and patient area with 34 chairs and one bed for administration of chemotherapeutic agents (Fig. 1). Patients attend the center according to their allocated time slots throughout the day. Educational materials on hand hygiene practices are prominently displayed for patients. Additionally, alcohol-based hand rub (AHR) bottles are available in the reception area, waiting area, and chemotherapy administrative area for use by both patients and staff.

### Hand hygiene in chemotherapy day center

The study was divided into three phases. In the first phase (1 April to 31 May 2023), Infection Control Nurse (ICN) observed hand hygiene compliance among patients and HCWs at the entrance and reception area for 20 min daily upon the center’s opening at 9:00 AM on working days. The ICN observed whether patients practiced hand hygiene by using the AHR available in the reception area. Additionally, the ICN observed whether HCWs (including nurses and support staff) practiced hand hygiene before and after measuring patients’ temperature and blood pressure during the registration process.

In the second phase (1 June to 31 July 2023), 53 pressure sensors were installed at the bottom of 500-ml bottles of AHR at designated sites in the chemotherapy day center (Fig. 1). Each pump of the alcohol-filled bottle generated pressure, which was counted by the pressure sensor as one usage. HCWs were advised to use the sensor-equipped AHR bottles instead of their pocket-sized AHR during the study period. The ICN continued to observe hand hygiene compliance among patients and HCWs in the morning, similar to phase one. Furthermore, the ICN collected data from the pressure sensors through connection to the smartphone in the evening to analyze the frequency of hand hygiene practices during daytime hours.

In the third phase (1 August to 30 September 2023), a robot named Temi Medic (HandyRehab, Zunosaki Limited, Hong Kong SAR) was introduced to the chemotherapy day center. Temi Medic (hereafter referred to as Temi) is a versatile, open-platform robot featuring advanced artificial intelligence and voice interaction. It enhances patient care through telemedicine, robot-enabled tele-visits, and fall management, while also assisting in ward management through patient education and message broadcasting. In this study, Temi was programmed to promote hand hygiene by broadcasting educational videos along a designated route inside the center. From 9:00 AM to 10:00 AM, Temi remained at the entrance as patients began registering at the reception area. Then, Temi started moving inside the center during three different time intervals: 11:00 AM to 12:00 PM, 1:00 PM to 2:00 PM, and 3:00 PM to 4:00 PM. During these intervals, Temi repeatedly stopped at seven designated spots, including the waiting areas, reception area, and chemotherapy administration area, following a specified path and broadcasting 5-min hand hygiene promotion videos at these locations (Fig. 1). The content of educational materials delivered by TEMI is shown in the Supplementary File. The ICN collected data similar to phase 2 to analyze whether the frequency of hand hygiene among patients and HCWs increased with the robot’s presence and promotional activities.

### Statistical analysis

The Fisher’s exact test, Chi-Square test, Student’s *t-*test, and One-way ANOVA were used as appropriate. A *p*-value of < 0.05 was considered statistically significant.

## Results

### Attendance in the chemotherapy day center

From 1 April to 30 September 2023, a total of 11,760 episodes involving 2580 patients attending the chemotherapy day center were recorded. The median age of these patients was 63 years (range: 19 to 96 years). There were 3694 episodes (1660 new patients) in phase 1 (38 working days), 3846 episodes (510 of 1684 patients as new cases) in phase 2 (41 working days), and 4220 episodes (410 of 1682 patients as new cases) in phase 3 (42 working days).

### Hand hygiene practices before and after utilizing a robot for promotion

During the daily 20-min unobtrusive observations in the reception area in the morning, the ICN observed a significant increase in hand hygiene practices among patients, rising from phase 1 (0.2%, 1/583) and phase 2 (0.5%, 3/656) to phase 3 (5.0%, 33/654) (*p* < 0.001), when Temi was utilized to disseminate hand hygiene promotion videos in the chemotherapy center. In contrast, the observed hand hygiene compliance among HCWs showed no significant difference across the three phases, with an overall compliance rate of 74.1% (1341/1810).

During phases 2 and 3, the frequency of AHR use was monitored by pressure sensors. Among the 7 sensor-equipped AHR bottles designated for patient use (P1–P7), the mean count of AHR per 100 patient attendances per day (hereafter referred to as the mean count) significantly increased from phase 2 to phase 3 (35 ± 17 vs. 64 ± 24, *p* < 0.001) (Table 1), resulting in an 83% increase and a 95% confidence interval (95% CI) for the mean count difference of 23 to 35. Specifically, the mean count was significantly higher in P1–P5 (31 ± 16 vs. 55 ± 23, *p* < 0.001), which were located in the waiting areas, as well as in P6–P7 (4 ± 4 vs. 8 ± 5, *p* < 0.001), which were placed at the entrance of chemotherapy administration area. Similarly, for the 33 sensor-equipped AHR bottles (S1-S33) shared by both patients and HCWs, the mean count also significantly increased from 207 ± 104 to 267 ± 113 (*p* = 0.027), representing a 29% increase, with a 95% CI for the mean count difference of 8 to 112. There was no significant change in the mean count among the 13 sensor-equipped AHR bottles (H1–H13) designated for HCWs between phases 2 and 3 (Table 1). The mean count of H1–H13 was significantly higher than that of P1–P7 throughout phases 2 and 3 (214 ± 93 vs. 49 ± 25, *p* < 0.001), showing a 4.4-fold difference. The 95% CI for the mean count difference was 111 to 219.

**Table 1:** Hand hygiene monitoring in the chemotherapy day center at Queen Mary Hospital

	Phase 1^a^ (1 Apr to 31 May 2023)	Phase 2^b^ (1 Jun to 31 Jul 2023)	Phase 3^c^ (1 Aug to 30 Sep 2023)	*p* value
Working days	38	41	42	
Total number of attendances	3694	3846	4220	
Daily attendance (mean ± S.D.)	97 ± 9	94 ± 7	100 ± 12	0.010^d^
*HH among patients observed by ICN*^*e*^				
No. of patients observed at reception	583 / 3694 (15.8%)	656 / 3846 (17.1%)	654 / 4220 (15.5%)	
No. (%) of patients practiced HH at reception	1 / 583 (0.2%)	3 / 656 (0.5%)	33 / 654 (5.0%)	< 0.001^f^
*HH among HCWs observed by ICN*^*e*^				
Total number of observed HH opportunity	578	645	587	
Observed HH opportunity per day (mean ± S.D.)	15 ± 7	16 ± 3	14 ± 6	0.360^d^
Observed HH compliance per day (mean ± S.D.)	67.3 ± 13.2	68.4 ± 10.3	72.4 ± 10.4	0.085^d^
*HH monitored by pressure sensor*^*g*^				
No. of AHR with sensor used by patients	NA	7	7	
Count of AHR used by patients per 100 patient attendances per day (mean ± S.D.)	NA	35 ± 17	64 ± 24	< 0.001^h^
No. of AHR with sensor used by both patients and HCWs	NA	33	33	
Count of AHR used by both patients and HCWs per 100 patient attendances per day (mean ± S.D.)	NA	207 ± 104	267 ± 113	0.027^h^
No. of AHR with sensor used by HCWs	NA	13	13	
Count of AHR used by HCWs per 100 patient attendances per day (mean ± S.D.)	NA	226 ± 95	201 ± 88	0.218^h^

AHR, alcohol-based hand rub; HCWs, healthcare workers; HH, hand hygiene; ICN, infection control nurse; NA, not applicable; S.D, standard deviation

^a^Baseline period before the installation of pressure sensors for the alcohol-based hand rub in the chemotherapy day center

^b^Installation of 53 pressure sensors for the alcohol-based hand rub in the chemotherapy day center, prior to using the robot for hand hygiene promotion

^c^Installation of 53 pressure sensors for the alcohol-based hand rub in the chemotherapy day center, along with the use of a robot for hand hygiene promotion

^d^By one-way ANOVA

^e^A 20-min session of unobtrusive observation was conducted by the infection control nurse in the reception area of the chemotherapy day center on each working day. To assess hand hygiene practices among patients, the infection control nurse observed whether patients used the alcohol-based hand rub available at the registration counter in the reception area. For healthcare workers, the nurse observed whether they performed hand hygiene according to the World Health Organization recommendation

^f^By Fisher’s exact test

^g^Pressure sensors were installed at the bottom of 500-ml bottles of alcohol-based hand rub in the reception, waiting, and patient area of the center. Each pump of the alcohol-filled bottle generated pressure, which was counted as one usage

^h^By Student’s *t*-test

Temi also carried a bottle of AHR (the 54th pressure sensor-equipped AHR) to facilitate hand hygiene practices among patients during four 1-h intervals per day in phase 3, resulting in a mean count of 7 ± 6. Compared to another four 1-h intervals when Temi was not actively operating in the center, there was no significant difference in the mean counts collected with or without Temi serving: from P1–P7 (30 ± 15 vs. 25 ± 14, *p* = 0.106) and S1-S33 (112 ± 62 vs. 116 ± 63, *p* = 0.267) during phase 3.

## Discussion

In this study, the introduction of the robot Temi in the Chemotherapy Day Center demonstrated an 83% increase in hand hygiene practices among patients, suggesting that social robots can effectively engage individuals and serve as reminders for hygiene practices. Our findings align with previous studies using the humanoid robots DAVE, which achieved a 29% improvement in hand hygiene compliance in a hospital setting [3]. Hand hygiene compliance poses a significant challenge due to the intrinsic nature of human behavior, influenced by various factors, including biology, environment, education, and culture [5]. A crisis, such as the COVID-19 pandemic, can push hand hygiene compliance among HCWs to 100% [6]. However, the complexity of human behavior makes it difficult to motivate HCWs to consistently comply with hand hygiene practices. During our daily audits by the ICN throughout the study period, the overall hand hygiene compliance among HCWs in the day center was 74%, despite our active advocacy for the WHO’s initiative on hand hygiene using AHR as a key strategy to prevent healthcare-associated infections [5, 7].

To address this, we extended hand hygiene promotion from HCWs to patients by implementing directly observed hand hygiene (DOHH) for conscious hospitalized patients before meals and medication rounds [8, 9]. These DOHH-based infection control measures not only minimized the incidence of nosocomial outbreaks caused by epidemiologically important pathogens [10], but also helped control the nosocomial transmission of vancomycin-resistant Enterococci [11] and multi-drug resistant *Acinetobacter baumannii* [12, 13]. Additionally, our previous study indicated that patients, particularly within the context of Chinese culture, tend to be introvert during hand hygiene empowerment initiatives [14]. This suggests that innovative technology is necessary for effective hand hygiene promotion.

The use of a robot to broadcast informative videos at strategic locations within the center has proven to be more effective than traditional reminder methods, such as informational signs or passive cues. Less than 1% of patients used AHR at the entrance and reception area during phases 1 and 2, as observed by the ICN. However, patients’ self-initiated use of AHR increased to 5% during phase 3 when Temi was in service. While this increase represents a positive trend, the overall uptake remains alarmingly low across all phases. This extremely low uptake underscores a significant concern: patients may not be alerted about the benefits of using AHR, which is particularly concerning given that this study was conducted after the peak of the COVID-19 pandemic. This lack of awareness may suggest that patients may not receive adequate information or counseling regarding the critical importance of hand hygiene practices. Therefore, it is vital to implement better communication strategies to educate patients about hand hygiene. Although the observed episodes of hand hygiene practices by patients remained low, this still reflects the impact of Temi. Notably, only 410 (24%) of the 1682 patients were new to the center; patients with repeated visits may have become more aware of the AHR at the entrance or in the reception area, either through memory or reminders from Temi on-site during phase 3.

While the ICN is required to observe hand hygiene practices for both patients and HCWs, the observed episodes in both categories may be lower than the actual number. The pressure sensor data may provide a more accurate report of AHR use frequency, serving as a surrogate marker for hand hygiene practices. By adjusting the absolute counts from each AHR bottle to the mean count per 100 attendances per day, the mean counts significantly increased by 83% among the AHR bottles designated for patient use from phase 2 to phase 3. Similarly, the mean counts also significantly increased by 29% among the AHR bottles shared by both patients and HCWs. The increase in the mean counts of the shared AHR bottles may likely be attributed to patient use, as there was no significant difference in the mean counts of the AHR bottles exclusively used by HCWs.

The non-significant change in the mean count among AHR bottles used by HCWs may be attributed to the educational materials delivered by TEMI primarily focusing on patients rather than HCWs. Alternatively, it could indicate that HCWs are already practicing good hand hygiene due to established protocols. However, this raises questions about the potential for robotic interventions to further enhance compliance among staff. At times, new intervention initiatives may not be perceived as engaging by professional staff. For instance, our promotion of influenza vaccination through social media resulted in a significant increase in the vaccine update rate among non-professional staff, but not among professional staff [15]. Future studies could explore targeted strategies to engage HCWs more effectively, perhaps by integrating the robot into training sessions or feedback mechanisms [16].

The lack of significant differences in AHR usage during the time intervals when Temi was actively promoting hand hygiene versus when it was not may imply that behavior change occurs, at least in the short term. While the robot did increase hand hygiene practices among patients during its operational hours, we did not observe a significant drop in hand hygiene practices during Temi’s non-operating hours within the same day. However, the long-term impact of such interventions remains to be seen. It is essential to investigate whether the initial increase in hand hygiene practices can be maintained over time, as repeated exposure to the robot may lead to habituation or decreased effectiveness [17].

This study has several limitations that should be considered. First, the short evaluation period limits our ability to assess the long-term sustainability of the observed increases in hand hygiene compliance. Second, with the study conducted in a single chemotherapy day center, the findings may not be generalizable to other healthcare settings. Third, reliance on AHR usage as a surrogate marker may not fully reflect actual hand hygiene compliance according to WHO recommendations among HCWs [5]. However, the mean count of sensor-equipped AHR may monitor the trend of hand hygiene practices among patients and HCWs in a longitudinal and unobtrusive manner. Finally, the lack of patient-reported outcomes restricts our understanding of the robot’s acceptability. Addressing these limitations in future research will be crucial for validating the effectiveness of robotic interventions in promoting hand hygiene compliance.

## Conclusions

The introduction of the Temi robot notably enhanced hand hygiene practices among patients in the chemotherapy day center, demonstrating the effectiveness of robotic interventions in promoting these practices. While healthcare workers maintained stable compliance, the need for additional engagement strategies remains evident. Future research should focus on the long-term sustainability and generalizability of these findings, as well as exploring patient perceptions of robotic support. Overall, this study underscores the potential of integrating innovative technology to improve infection prevention and control in healthcare settings.

## Supplementary Information

Additional file 1.

## Data Availability

The datasets generated for this study will be made available in anonymized form from the corresponding author upon reasonable request.
