# Supplementary material for: Promoting hand hygiene in a chemotherapy day center: the role of a robot
Source: Antimicrob Resist Infect Control. 2024 Dec 21;13:154. doi: 10.1186/s13756-024-01510-5 (PMC11663329; doi:10.1186/s13756-024-01510-5)
Supplement: Supplementary file 1 — Additional file 1. [file 13756_2024_1510_MOESM1_ESM.pdf]

Supplementary File

The script broadcast by TEMI in Cantonese (Chinese) at the entrance reception area from 9:00 AM to 10:00 AM is as follows:

“進入日間化療中心前，請記得用酒精搓手液搓手啊！手部衛生有效預防細菌傳播，各位記得搓手啊。如果手上冇明顯污糟，就可以用酒精搓手液潔手。個人衛生要做好，潔手梗係要做到！對抗細菌病毒，潔手好緊要㗎！入面有好多酒精搓手液俾大家用㗎。”

The English translation of the above script is as follows:

"Before entering the Chemotherapy Day Center, please remember to use hand sanitizer! Hand hygiene is an effective way to prevent the spread of bacteria, so everyone, please remember to sanitize your hands. If your hands are not visibly dirty, you can use hand sanitizer to clean them. Good personal hygiene is essential, and hand cleaning must be done! To fight bacteria and viruses, hand hygiene is very important! There are plenty of hand sanitizers available inside for everyone to use."

Five videos played by TEMI at designated spots inside the Chemotherapy Day Center were produced by the Hospital Authority (HA) and the Centre for Health Protection (CHP), Department of Health in Hong Kong, and are available on internet The links, accessed on November 19, 2024, are shown below:

CHP – 2022年手部衛生日 Hand hygiene animation 2022:

https://www.youtube.com/watch?v=CxDvQNer_TM

CHP - 使用酒精搓手液潔手:

https://www.youtube.com/watch?v=YGSbWriRl6U

CHP - 提一提 冇問題（醫院篇）:

https://www.youtube.com/watch?v=YhcosYemtxA

HA - 提一提‧無問題:

https://video.ha.org.hk/video?vid=318&lang=zh-HK

HA - 病人手衛生 Patient Hand Hygiene:

https://www.youtube.com/watch?v=f1Pk-xSKrEE
